# Uncertain About Uncertainty: How Qualitative Expressions of Forecaster Confidence Impact Decision-Making With Uncertainty Visualizations

**DOI:** 10.3389/fpsyg.2020.579267

**Published:** 2021-01-22

**Authors:** Lace M. K. Padilla, Maia Powell, Matthew Kay, Jessica Hullman

**Affiliations:** ^1^ Spatial Perception, Applied Cognition and Education (SPACE) Lab, Cognitive and Information Sciences, University of California Merced, Merced, CA, United States; ^2^ Applied Mathematics, University of California Merced, Merced, CA, United States; ^3^ Midwest Uncertainty Collective (MU Collective), Computer Science and Engineering, Northwestern University, Evanston, IL, United States

**Keywords:** uncertainty, visualization, cognition, direct uncertainty, indirect uncertainty, aleatory, quantile dotplots, decision-making

## Abstract

When forecasting events, multiple types of uncertainty are often inherently present in the modeling process. Various uncertainty typologies exist, and each type of uncertainty has different implications a scientist might want to convey. In this work, we focus on one type of distinction between *direct quantitative uncertainty* and *indirect qualitative uncertainty*. Direct quantitative uncertainty describes uncertainty about facts, numbers, and hypotheses that can be communicated in absolute quantitative forms such as probability distributions or confidence intervals. Indirect qualitative uncertainty describes the quality of knowledge concerning how effectively facts, numbers, or hypotheses represent reality, such as evidence confidence scales proposed by the Intergovernmental Panel on Climate Change. A large body of research demonstrates that both experts and novices have difficulty reasoning with quantitative uncertainty, and visualizations of uncertainty can help with such traditionally challenging concepts. However, the question of if, and how, people may reason with multiple types of uncertainty associated with a forecast remains largely unexplored. In this series of studies, we seek to understand if individuals can integrate indirect uncertainty about how “good” a model is (operationalized as a qualitative expression of forecaster confidence) with quantified uncertainty in a prediction (operationalized as a quantile dotplot visualization of a predicted distribution). Our first study results suggest that participants utilize both direct quantitative uncertainty and indirect qualitative uncertainty when conveyed as quantile dotplots and forecaster confidence. In manipulations where forecasters were less sure about their prediction, participants made more conservative judgments. In our second study, we varied the amount of quantified uncertainty (in the form of the SD of the visualized distributions) to examine how participants’ decisions changed under different combinations of quantified uncertainty (variance) and qualitative uncertainty (low, medium, and high forecaster confidence). The second study results suggest that participants updated their judgments in the direction predicted by both qualitative confidence information (e.g., becoming more conservative when the forecaster confidence is low) and quantitative uncertainty (e.g., becoming more conservative when the variance is increased). Based on the findings from both experiments, we recommend that forecasters present qualitative expressions of model confidence whenever possible alongside quantified uncertainty.

## Introduction

Various types of uncertainty are inherent in most modeling procedures (Pang et al., 1997), particularly in weather forecasting models where *direct quantitative uncertainty* (e.g., 25% chance of rain) and *indirect qualitative uncertainty* concerning the accuracy of the forecast are present. Direct quantitative uncertainty expresses uncertainty about facts, numbers, and hypotheses in absolute quantitative forms such as probability distributions or confidence intervals (van der Bles et al., 2019). Uncertainty visualization has historically focused on developing and testing methods for communicating direct quantitative uncertainty (for review see, Padilla et al., 2020). As researchers van der Bles et al. (2019) acknowledge that less work has focused on other types of uncertainty, including indirect qualitative uncertainty, which expresses the quality of knowledge about how effectively the facts, numbers, or hypotheses represent reality. Each modeling or forecast procedure is a tool for expressing real-world data, and therefore, some models are more representative of reality than others (McElreath, 2016). For example, a model may miss important data, calibrate poorly, or have incorrect assumptions, all of which may decrease a model’s accuracy. In an ideal setting, where all relevant data and relationships are known, there would be no need for indirect qualitative uncertainty. However, in many domains, this ideal cannot be attained; thus, indirect qualitative uncertainty is widely used for expressing experts’ subjective evaluation of model and forecast quality, such as the evidence confidence levels proposed by the Intergovernmental Panel on Climate Change (IPCC; Stocker et al., 2013; for a review of scientific evidence rating systems, see also West et al., 2002).

Without a clear understanding of how people conceptualize multiple types of uncertainty, we cannot know how people will respond when presented with qualitative uncertainty concerning the accuracy of a model. Prior work indicates that even reasoning with only quantified uncertainty – without qualitative uncertainty included – can be challenging for both novices and trained experts (Belia et al., 2005). In one study, researchers found that professionals (e.g., professors, researchers, and physicians) in psychology, behavioral neuroscience, and medicine misunderstood how 95% confidence intervals, shown with error bars, relate to statistical significance (Belia et al., 2005). These results are concerning, because many scientific publications in the professionals’ respective disciplines use error bars to illustrate statistical significance. Consequently, reasoning with direct and indirect uncertainty simultaneously will likely be challenging. Emerging research in data visualization has developed promising new ways to communicate traditional challenging statistical concepts, such as quantified uncertainty, more intuitively (for review, see Padilla et al., 2020). However, more work is needed to determine if the advancements in data visualization support reasoning enough to help people conceptualize indirect and direct uncertainty when presented together.

Given that little is known about how people reason with various types of uncertainty, many data communicators are hesitant to represent uncertainty in their science. In a survey of 90 visualization authors, Hullman (2019) found that authors (e.g., data scientists, journalists, visualization designers, and science communicators) are reluctant to visualize quantified uncertainty, perceiving that it may lessen the credibility and effectiveness of their results and that audiences may find it off-putting or overwhelming. Scientists may also fear that communicating the uncertainty in their models invites criticism, indicates incompetence, or decreases trust in their science (Fischhoff, 2012; Gustafson and Rice, 2019).

**Figure 1:**
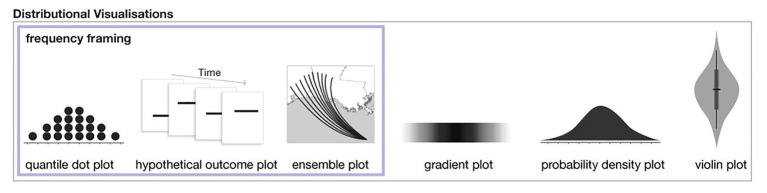
A subset of distributional uncertainty visualization techniques as described in Padilla et al. (2020). The three methods on the left also utilize frequency framing.

Whereas both direct and indirect uncertainties are prevalent in most forecast models, the question of if, and how, people reason with multiple types of uncertainty from a single forecast remains mostly unexplored. The goal of the current work is to examine the how people make decisions with direct and indirect uncertainties to gain insights into how we conceptualize various types of uncertainties and their combinations. In the presented series of studies, we use a visualization technique entitled *quantile dotplots* to communicate direct uncertainty, which is a modern, empirically validated method for communicating direct uncertainty in the form of distributions (see Figure 1; Kay et al., 2016; Fernandes et al., 2018). Participants in the current series of studies were tasked with completing a resource allocation judgment using quantile dotplots that display a forecast for predicted low nighttime temperatures. In some conditions, participants were also provided with indirect uncertainty in the form of forecaster confidence in the nighttime low-temperature prediction. Within the context of a resource allocation judgment, we tested if people can incorporate information about forecasts and forecaster confidence in their decisions. This study provides the first insights into how people conceptualize both direct and indirect uncertainty within a single forecast using best practices in information communication.

### Background

Scholars have proposed numerous uncertainty typologies to classify forms of uncertainty (e.g., van Asselt and Rotmans, 2002; Walker et al., 2003; Morgan et al., 2009; Spiegelhalter, 2017; for a recent review, see van der Bles et al., 2019). Researchers frequently recognize two categories of uncertainty but differ on the terms they use to describe these. One consists of uncertainty that can be directly quantified: e.g., *epistemic* (Der Kiureghian and Ditlevsen, 2009), *error* (Goodman and Nguyen, 1999), *risk* (Knight, 2012), and *first-order* uncertainty (Bach et al., 2009). These types of uncertainties can be characterized by mathematical expressions that denote error or variability in measurements, such as probability distribution functions (PDFs) or standard error (SE), which can be used to make predictions about future events. For example, the likelihood of rain tomorrow can be expressed in probabilistic terms that can be calculated directly (i.e., 25% chance of rain). In line with the terminology proposed by van der Bles et al. (2019), we will refer to absolute quantitative forms of uncertainties as direct quantitative uncertainty. The second category of uncertainty is comprised of those uncertainties that cannot be quantified directly, e.g., *ontological* (Spiegelhalter, 2017), *ambiguity or true uncertainty* (Knight, 2012), and *second-order* uncertainty (Bach et al., 2009). Indirect uncertainty exists when a model has variability or error that the modeler cannot foresee or quantify, possibly due to unknown amounts or levels of missing data, or unidentifiable error that enters the modeling pipeline (Pang et al., 1997), among other causes. For example, the accuracy of a rain forecast model for tomorrow is higher than the accuracy of a model that predicts the likelihood of rain 2 years from now. By definition, such unquantifiable uncertainties can be expressed only in subjective forms, as in the IPCC forecaster confidence ratings (Stocker et al., 2013). In line with van der Bles et al. (2019), we will refer to unquantifiable uncertainty as indirect qualitative uncertainty. In particular, this work focuses on expressions of the quality of knowledge concerning how accurately the facts, numbers, or hypotheses represent reality (van der Bles et al., 2019).

Even though uncertainty is inherent in all future forecasts, most of the public has difficulty reasoning with conventional forms of uncertainty communication such as standard probability formats, which are expressed with terms such as *probability*, *likelihood*, *chance*, or *odds*. A seminal research program by Kahneman and Tversky (e.g., Tversky and Kahneman, 1974; Kahneman and Tversky, 1977, 1979, 1982, 1984) systematically documented the countless ways that people rely on heuristics, or rules of thumb, to make judgments with probabilities rather than using the mathematically correct solution. Evidence that people made predictable errors when reasoning with probabilities led some researchers to propose that human logic is systematically flawed (for a critical discussion of this debate, see Vranas, 2000). Subsequent work by Gigerenzer (1996) suggests that decisions may appear to be flawed when people are presented with overly confusing information that can influence their judgments in submathematically optimal ways. Gigerenzer et al. proposed that probabilistic decisions become more intuitive when the format of an expression more naturally corresponds to how people experience probability throughout their lives (e.g., changing 10% to 1 out of 10; e.g., Gigerenzer and Hoffrage, 1995; Gigerenzer, 1996; Hoffrage and Gigerenzer, 1998). Numerous studies support the hypothesis that using a *frequency framing* of numeric expressions improves probabilistic judgments (for review and caveats, see Visschers et al., 2009). For example, one study found that when gynecologists were presented with information about breast cancer screenings using probabilities (e.g., 1% probability of breast cancer in the population; mammograms are 90% accurate; the likelihood of a false-positive is 9%), 790 of 1,000 incorrectly answered a question about a patient’s breast cancer odds. However, after training on how to convert probabilities to frequencies (e.g., 1 of 100 women in the population have breast cancer), 870 of the 1,000 gynecologists were able to correctly respond to a question about a patient’s breast cancer odds (Hoffrage and Gigerenzer, 1998).

A growing body of research finds that visualizations that show distributional information in frequency framing can improve accuracy and memory compared to visualizations that show only probability distributions and/or summary statistics (e.g., Kay et al., 2016; Ruginski et al., 2016; Hullman et al., 2017; Padilla et al., 2017; Fernandes et al., 2018; Kale et al., 2020; see examples of distributional visualizations and those that use frequency framing in Figure 1). A reliable finding across uncertainty visualization research is that static interval plots, such as the ubiquitous 95% confidence interval, can lead to errors and biases (e.g., Belia et al., 2005; Joslyn and LeClerc, 2012; Padilla et al., 2017). Many studies find that increasing the expressiveness of an interval plot by displaying distributional information can improve performance, for example, with quantile dot plots (Kay et al., 2016; Fernandes et al., 2018; Kale et al., 2020), hypothetical outcome plots (Hullman et al., 2015; Kale et al., 2018), ensemble plots (Ruginski et al., 2016; Padilla et al., 2017), gradient plots, and violin plots (Correll and Gleicher, 2014). Among the visualizations that show distributional information, those that include frequency framing, specifically quantile dot plots and hypothetical outcome plots, have been found to outperform other distributional visualizations (Hullman et al., 2015; Kay et al., 2016; Fernandes et al., 2018; Kale et al., 2018, 2020). Best practices in uncertainty visualization suggest distributional information be displayed using frequency framing when possible and in a way that does not allow the viewer to fixate on summary information such as a mean (Kale et al., 2020). For a full review of modern uncertainty visualization techniques and theory, see Padilla et al. (2020).

**Figure 2:**
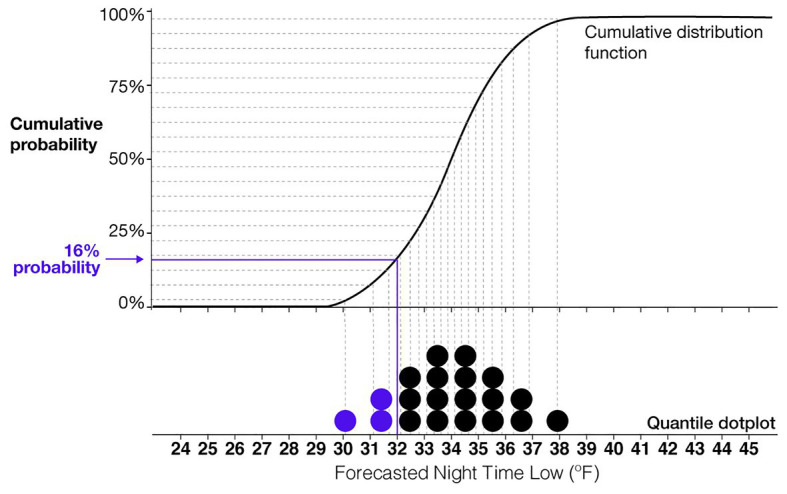
An illustration of the relationship between a quantile dotplot and cumulative distribution function. This example shows a normal distribution of a forecasted nighttime low temperature with a mean of 34°F and SD of 2°F. Each dot represents a 5% probability.

One of the most consistently high performing uncertainty visualizations is the quantile dotplot. Quantile dotplots use stacked dots to represent a probability distribution (see Figure 2). Each dot is a quantile of the distribution, such that each dot represents the same probabilistic value. Figure 2 shows a cumulative distribution function for a normal distribution of forecasted nighttime low temperatures and the corresponding quantile dotplot that represents the distribution. In this example, each dot represents a 5% probability. The viewer can count the number of dots in a given range to determine the probability of the nightly low temperature falling within that range. In several empirical studies, researchers have found that quantile dotplots improve memory of distributional information and lead to more consistent probability estimates compared to probability density plots (Kay et al., 2016; Hullman et al., 2017; Kale et al., 2020). More studies have found that quantile dotplots outperform interval plots, density plots, and textural descriptions of uncertainty for decisions with risk (Fernandes et al., 2018). When viewers need to mentally combine multiple estimates of uncertainty from different sources, researchers have found that quantile dotplots and density plots improve participants’ judgments compared to confidence intervals and point estimates (Greis et al., 2018). Quantile dotplots are a promising visualization technique that utilizes the benefits of frequency framing and incorporates the best practices in visualization research (Padilla et al., 2020).

In the context of indirect qualitative uncertainty, a large amount of work has been dedicated to examining how people reason with linguistic expressions of experts’ interpretations of scientific accuracy (van der Bles et al., 2019). For example, West et al. (2002) offer a review of over 19 grading systems that can be used to evaluate the strength of a body of evidence in a clinical context using expert ratings of quality, quantity, and consistency of scientific evidence. Other systems include categorical ratings of the quality of evidence such as *high*, *moderate*, *low*, and *very low* (Balshem et al., 2011; also see Schneider and Moss, 1999; Stocker et al., 2013). Nevertheless, a long history of research demonstrates that significant variability exists in the assumed probabilistic values attributed to linguistic uncertainty expressions (for review, see O’Hagan et al., 2006), which is highly dependent on individual differences and the context of the uncertainty (Budescu et al., 2009, 2012).

To date, no research has empirically tested how people reason with both direct and indirect uncertainty information about a single forecast. However, researchers have explored approaches for generating forecasts with multiple occurrences of uncertainty. A number of these approaches have implemented weather forecasting using natural language generation (NLG) and addressed ambiguity in language. One study produced pollen forecasts for Scotland through spatiotemporal analysis in an effort to effectively forecast information (Turner et al., 2006), and another generated short-term weather forecasts in the form of natural language texts using fuzzy procedures to model imprecise descriptions (Ramos-Soto et al., 2014). Other related work advanced the analysis of protoforms (structures for fuzzy quantified sentences) to account for the imprecision of linguistic descriptors of numerical data (Ramos-Soto and Martin-Rodilla, 2019). Researchers have also demonstrated the use of textual descriptions of forecasts along with forecast icons such as rain, cloud, or sun icons (e.g., Ramos-Soto et al., 2015). To our knowledge, researchers have not considered how people conceptualized direct visualizations of uncertainty along with indirect textural descriptions of uncertainty.

Additional research has also sought to computationally integrate indirect expert estimates of scientific quality with direct uncertainty expressions (e.g., Harmel et al., 2010; Turner et al., 2012; Darvishian et al., 2014; Rhodes et al., 2020). Turner et al. (2012) developed a method for constructing prior distributions based on expert opinions that can be incorporated into direct quantitative uncertainty to reflect varying levels of quality, entitled *bias-adjusting*. Scholars have applied the process of bias-adjusting estimates in various applications, notably within meta-analyses of clinical trials (e.g., Turner et al., 2012; Darvishian et al., 2014; Rhodes et al., 2020). Other methods for computationally integrating direct and indirect uncertainty include correction factors based on predictions of model fit (Harmel et al., 2010). This work is promising, but researchers have not examined how people reason with bias-adjusted estimates, resulting in no clear guidance as to when such techniques are appropriate for a general audience. Further, when direct and indirect uncertainty are computationally combined and communicated as a summary, transparency is reduced, which may negatively impact trust (O’Neill, 2012, 2018; van der Bles et al., 2020). The present research focuses on determining how people conceptualize direct and indirect uncertainties that are communicated independently about a single forecast. The results of our work can be used as a baseline to determine if people conceptualize computationally aggregated direct and indirect uncertainties in a manner similar to the way they would with the two shown independently.

Researchers have also shown significant interest in examining how people mentally combine subjective expert uncertainty estimates from multiple sources (e.g., Wallsten et al., 1997; Clemen and Winkler, 1999; Ariely et al., 2000; Budescu, 2005) and how to computationally aggregate estimates from multiple experts (e.g., Fan et al., 2019; Han and Budescu, 2019). When people think a phenomenon has uncertainty, they will commonly look at multiple sources of information to reduce their perceived uncertainty (Greis et al., 2017). For example, people may seek a second opinion about medical procedures or look to multiple weather forecasts to determine the level of agreement between forecasters. When asked to mentally aggregate estimates from multiple experts, they commonly mentally average the estimates (Budescu, 2005). Further visualizations that show fine-grained uncertainty in an estimate, such as quantile dotplots, can improve how people mentally integrate uncertainty information from multiple sources (Greis et al., 2018).

Although the research described above systematically reveals how people reason with uncertainty from multiple sources and the variability within a single source, researchers are less clear about how these findings generalize to multiple types of uncertainty from the same source (e.g., direct quantitative and indirect qualitative uncertainty from a single source). For example, a simple solution for determining the most likely forecasted nighttime low temperature using two competing forecasts is to compute the average mean temperature from both forecasts, a strategy that people commonly use (Ariely et al., 2000; Budescu, 2005). However, we have no such obvious way to mentally combine a forecasted nighttime low temperature with a subjective estimate of the forecaster’s confidence, because indirect uncertainty that is expressed as forecaster confidence does not have a defined value by definition. Given the complexity and variability within the interpretation of indirect uncertainty, people may use many different heuristic strategies when trying to reason with both direct and indirect uncertainty. The goal of the current work is to examine the strategies that people use when reasoning with direct and indirect uncertainty to provide scientists with practical advice on how to effectively communicate the uncertainty in their science.

### Overview of Experiments

As a first step in understanding how people reason with multiple types of uncertainty, we present a series of studies that aim to understand if individuals can integrate uncertainty about how “good” a model is (operationalized as an expression of forecaster confidence) with quantified uncertainty in a prediction (operationalized as a quantile dotplot visualization of a predicted distribution). In Experiment 1, we utilized quantile dotplots as an effective method to communicate direct uncertainty, and we selected forecaster confidence ratings as an imperfect but commonly used pragmatic method for communicating indirect uncertainty. We tested how people reason with both direct and indirect uncertainty in the context of a resource allocation judgment modeled after an experiment designed by Joslyn and LeClerc (2012). In the original study, participants were shown various forms of information about low nighttime temperatures and tasked with deciding to spend funds from a virtual budget to salt the roads if they believed the temperature would drop below freezing. If the participants failed to salt the roads when the road froze overnight, they were penalized by losing funds from a virtual budget. Joslyn and LeClerc (2012) found that the participants presented with a likelihood estimate of the temperatures dropping below 32°F made more accurate decisions and trusted the forecast more than participants presented with only a single estimate of the nighttime low temperature.

In the present studies, we adapted the context used in Joslyn and LeClerc (2012) to mimic reports from our colleagues at a large nongovernmental organization (NGO), in which they described cases where cold temperatures and snow forecasts were used to determine if emergency aid should be sent to alpaca farmers in Peru. In July of 2016, the government of Peru declared a state of emergency for regions where alpacas were raised after tens of thousands of alpacas died due to freezing temperatures (Hersher, 2016). The loss of livestock was devastating to the farmers in the affected regions of Peru, because exporting alpaca wool is the region’s primary source of income (Hersher, 2016). In 2016, NGOs had to decide when to provide aid to alpaca farmers, including cold-protective blankets and nutrition for the alpacas, since the area had experienced widespread crop loss as well. In 2013, alpaca farms experienced a similarly devastating cold spell that killed thousands of alpacas (Gallas, 2013; Hersher, 2016). Due to climate change, forecasters predict that areas like Peru will experience considerable fluctuations in temperature, resulting in more extreme temperatures (UN, 2020). Consistent with real-world judgments that NGOs made in 2016, participants were tasked with deciding to issue or withhold emergency aid for alpaca farmers in Peru based on distributional information about forecasted nighttime low temperatures and forecaster confidence in the accuracy of the forecast.

In Experiment 1, the goal was to test if descriptions of forecaster confidence in the accuracy of a forecast influenced participants’ decisions to issue or withhold emergency aid. In the instructions, participants were told that some remote areas of Peru were more challenging to forecast than others and that forecaster confidence reflects these differences. The primary manipulation of Experiment 1 was incorporating the level of forecaster confidence that accompanied one block of trial forecasts (High, Medium, and Low forecaster confidence).

**Figure 3:**
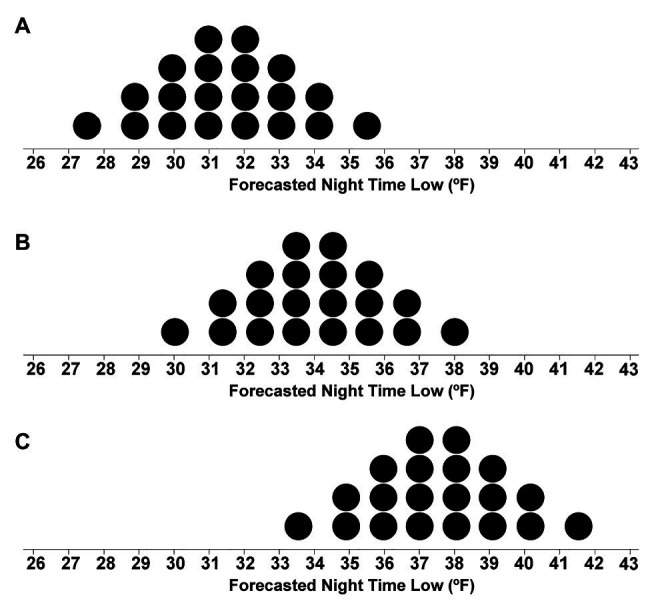
Stimuli depicting dotplot distributions with means of 31.5°F **(A)**, 34°F **(B)**, and 37°F **(C)**.

We visualized the nighttime low-temperature forecasts using quantile dotplots with the same variability (Medium-variance: SD of 2°F) but different mean temperatures for each forecast (ranging from 31.5 to 37°F, see Figure 3). By manipulating the mean temperature of the forecasts, we were able to test at which temperature participants switched from giving to withholding aid. As with real weather forecasts, in some cases, an unfavorable outcome is more likely than not to happen, and anticipatory action should be taken to minimize adverse outcomes. Similarly, our design presented participants with situations in which it was reasonably apparent that the temperature would likely be below freezing (Figure 3A with a mean of 31.5°F) and other cases in which it was clear the temperature would likely stay above freezing (Figure 3C with a mean of 37°F).

The first experiment’s goal was to consider the values between the extremes to determine at which point people would switch from giving to withholding aid (e.g., their crossover temperatures). In the first block, participants were provided with no information about forecaster confidence to identify their baseline crossover temperatures. Then, in the second block, participants were provided with additional information about forecaster confidence, which allowed us to determine how their judgments changed in response to the inclusion of indirect uncertainty.

In Experiment 2, we sought to examine how participants would integrate various direct quantitative uncertainty levels with the forecaster confidence ratings. We manipulated quantitative uncertainty by changing the SD of the quantile dotplots so that participants saw forecasts with low- (SD of 1°F, referred to as Low-variance), medium- (SD of 2°F, referred to as Medium-variance), and high-variability (SD of 3°F, referred to as High-variance) crossed with three levels of forecaster confidence.

The results of both studies will provide insights into the nature of how people conceptualize and make decisions with multiple types of uncertainty, which will provide previously missing guidance for scientific communication standards.

## Materials and Methods

### Participants

The sample size was determined using a power analysis based on effect sizes reported in Savelli and Joslyn (2013), which indicated that 85 participants would be needed to detect a medium effect size (Cohen’s *d* = 0.39). We recognize differences between the stimuli and tasks used in Savelli and Joslyn (2013) and the current work. However, we based the current experiment on the task used by Savelli and Joslyn (2013), and, therefore, it is the closest prior work to the current study, which is why we utilized the effect size to determine the necessary power in the present experiment. In Experiment 1, participants were 90 individuals (female = 38, male = 51, prefer not to say = 1, mean age = 39, *SD* = 9.69) with Master Class status on Amazon’s Mechanical Turk with a greater than 90% approval rating. In Experiment 2, participants were 90 individuals (female = 36, male = 53, preferred to self-describe = 1, mean age = 38, *SD* = 10) with the same Amazon’s Mechanical Turk qualifications.

### Stimuli

For Experiment 1, the package *ggplot2* v. 3.3.0 (Wickham, 2009) within the programming language R (R Core Team, 2013) was used to generate 12 horizontal quantile dotplots with SD of 2°F and mean temperatures ranging from 31.5 to 37°F with a step size of 0.5°F (see Figure 3; for example, stimuli). A quantile dotplot represents a distribution where dots are quantiles of the distribution. In this case, each dot depicts a 5% probability, because there are 20 dots total. To determine the predicted probability of the nighttime low temperature being 32°F or lower, a viewer counts the dots located on and to the left of 32°F and multiply the number of dots by 5 (or divides by 20). We presented the stimuli using Qualtrics survey software (Qualtrics, LLC, 2014). We used 20 dots as researchers found that 20 and 50 dots can be effective for quantile dotplots (Fernandes et al., 2018).

**Figure 4:**
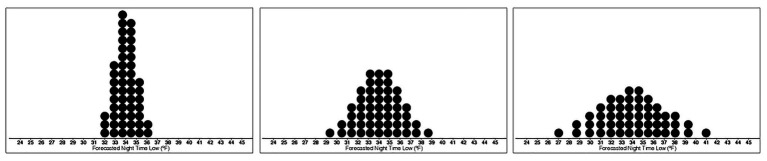
Stimuli depicting the base dotplot distributions with means of 34°F and Low- **(left)**, Medium- **(middle)**, and High-variance **(right)**.

For Experiment 2, the base images were created using the same procedure as in Experiment 1 but with SDs of 1, 2, and 3°F, with 50 dots (see Figure 4). Fifty dots were used instead of the previous 20 in Experiment 1 to reduce the visual skewing that occurred when fewer dots were used to display dotplots with SD of 1°F. The resulting dotplots had 12 mean values ranging from 31.5 to 37°F and three levels of variance, making a total of 36 stimuli.

### Design

**Figure 5:**
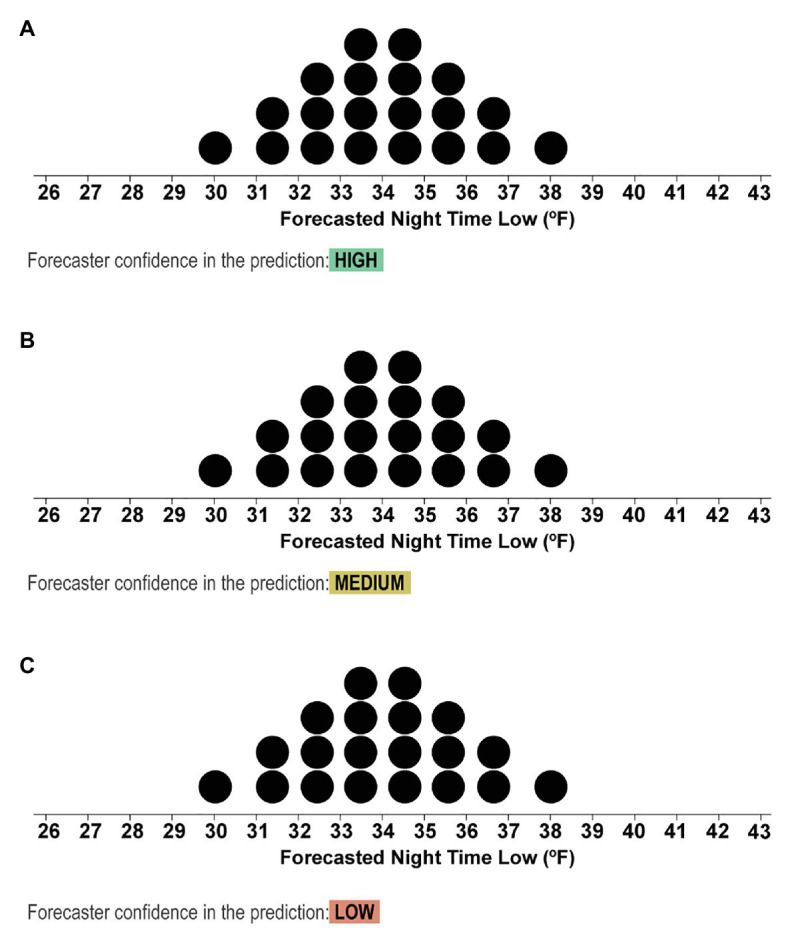
Stimuli from the second block depicting the dotplots with mean values of 31.5°F and text that indicated if the forecasters had High **(A)**, Medium **(B)**, or Low **(C)** confidence in the forecast.

In Experiment 1, we employed a blocked within-subjects design. The first block consisted of 12 randomly presented trials in which the mean temperature of the dotplots varied. The second block consisted of 36 trials with three variations on the trials used in the prior block. Under each of the forecasts, text was included that indicated if the forecaster had High, Medium, or Low confidence that the forecast represented the true distribution of low nighttime temperatures (see Figure 5). Thirty-six trials in the second block were presented in a randomized order. In sum, participants completed 48 trials.

For Experiment 2, the same blocked within-participant design was used as in Experiment 1, but we included the variance manipulation (Low-, Medium-, and High-variance) as an additional within-subjects manipulation. The first block consisted of 36 randomly presented trials in which the mean temperature of the dotplots and the variance were manipulated (within-participant: 12 mean temperatures and three levels of variance). The second block consisted of 108 trials with three variations on the trials used in the previous block. As in Experiment 1, under each of the forecasts, text was included that indicated if the forecaster had High, Medium, or Low confidence that the forecast represented the true distribution of low nighttime temperatures (within-participant manipulations: 12 mean temperatures, three levels of variance, and three levels of confidence).

### Procedure

For Experiment 1, after providing digital consent, participants were provided with instructions that described the task, context, and compensation. The task and compensation scales were adapted from prior work by Joslyn and LeClerc (2012). In the current experiment, participants were tasked with responding “yes” or “no” to issuing cold-protective blankets for alpacas based on quantile dotplots depicting the forecasted nighttime low temperature. The instructions for the task were as follows:

**Scenario**: *Alpacas may need blankets: Assume that you work at the Red Cross, and your job is to manage resources for farms in Peru. In previous years, alpacas have died in Peru from cold temperatures. Alpacas can typically withstand the cold unless the temperature drops below 32°F*.
**Budget**: *You are in charge of the Red Cross’s blanket budget, and it is your job to issue blankets to the alpacas when temperatures fall below 32°F, which will help them withstand the cold*.
**Budget Constraints**: *You have a budget for 48 days of $48,000. Purchasing and delivering blankets to farmers costs $1,000 (per night). If you fail to issue blankets to the farmers and the temperature drops below 32°F, it will cost $6,000 from your budget*.
**Task**: *In the experiment, you will be shown a nighttime temperature forecast like the one below. In the forecast, each dot represents a 1 out of 20 chance the nighttime low will be that temperature. You will be asked some questions about this forecast, including if you will issue blankets to the alpacas*.
**Compensation**: *Please respond to the best of your ability. You will receive an extra $0.15 cents for every $1,000 that you have in your budget at the end of 48 days*.

On every trial, participants were shown one quantile dotplot and text that reminded them, “Your job is to consider the forecast and determine if the nighttime low will drop below freezing, in which case you should issue blankets to the alpacas. Each dot in the forecast represents a 1 out of 20 chance.” After the first block (12 trials), participants then completed a second block (36 trials) in which confidence information about the forecast was also provided along with the dotplots. The following are the instructions for the second block:

*In this section of the experiment, you will be doing the same task as before, but you will be shown more information about the forecast models. Each of the forecasts will also include information about how confident the scientists are about the accuracy of the forecast. There are many factors that make the temperature difficult to predict, and scientists are more confident about some forecast models than others*.
**Example 1, high confidence**: *For example, see the forecast below and notice the information highlighted in green, which indicates that the scientists are highly confident that the forecast represents the true distribution of nighttime low temperatures*.
Example stimuli shown with high forecaster confidence as in Figure 5A.
**Example 2, low confidence**: *Now see the next forecast below. While the forecast may look the same, the scientists are less confident about the accuracy of this forecast because the prediction is for a remote area in Peru that the scientists have not forecasted before, and they do not have all the information they need to make an accurate forecast*.
Example stimuli shown with low forecaster confidence as in Figure 5C.

After the main experiment, participants were asked to rate how trustworthy they believed the forecasts with High-, Medium-, and Low-confidence were on a Likert scale ranging from 1 (not at all trustworthy) to 7 (completely trustworthy).

For Experiment 2, the procedure was the same between the two studies in all regards except that the overall budget was increased from $48,000 (for the 48 trials in Experiment 1) to $144,000 (for the 144 trials in Experiment 2). The instructions were updated to account for the different number of dots in the quantile dotplots and the increased number of trials and budget.

### Compensation

Participants in Experiment 1 were paid a base rate of $1.25 (in line with Illinois minimum wage laws) and provided additional performance-based compensation dictated by the remaining budget that each individual had at the end of the study. For the 48 total trials, individuals had $48,000, and if they decided to give blankets to the alpacas every day, they would have a remaining budget of $0. For every $1,000 left in their budget, participants received an extra $0.15, paid at the end of the study as a bonus. Participants were not given feedback until the end of the study regarding the remaining balance in their budget or on the accuracy of their judgments, and, therefore, this study did not measure learning effects. In Experiment 2, participants were paid a base rate of $6. For the 144 total trials, individuals had $144,000, and if they decided to issue blankets to the alpaca farmers every day, they would have a remaining budget of $0. For every $1,000 left in their budget, participants received an extra 5 cents paid at the end of the study as a bonus.

### Accuracy

Based on the $1,000 cost of providing blankets and the $6,000 penalty for withholding blankets, the optimal strategy would be to give blankets if the probability of the temperature being equal to or below 32°F is greater than 16.6% (e.g., 1,000/6,000 = 0.166). Consequently, the optimal crossover temperature (the forecast value at which one should switch from giving to not giving blankets) is the forecast value for which a Normal distribution with an SD of 2°F would have 16.6% of its probability mass below 32°F. That forecast value is 33.94°F: for *X*~Normal(33.94, 2), *P*(*X* < 32) = 16.6%. As an illustration of how this crossover point can be seen in a quantile dotplot (and the relationship between specific probabilities and quantile dotplots), we denoted 16% probability in the quantile dotplot in Figure 2. Given the forecast of 34°F in that figure (the approximate optimal crossover), we can see that roughly 16% of the probability mass is to the left of 32°F.

To compute participants’ crossover points, we derive the point at which there is a 50% probability they will give/not give blankets using a logistic regression model of their decisions. The response code is in the form of a Bernoulli distribution, where probabilities are the inverse logit (i.e., logistic) function of values derived from the latent linear predictor:

Blanket Decision ~ Bernoulli(*p*_i_)

$$p_{i}=logit^{-1}\left(\eta_{i}\right)$$

$$\eta_{i}=g\left(temp_{i};predictors_{i}\right)$$

where *g* is a function that makes the log odds of the person choosing to give blankets a linear function of the temperature, depending on the other predictors (e.g., condition and participant). Generically, this function follows the form:

$$g\left(temp;predictors\right)=intercept\left[predictors\right]+slope\left[predictors\right]\cdot temp$$

That is, each person in each condition may have a particular slope and intercept in this linear function. The crossover temperature for a given set of predictors, *t*(*predictors*), is then the point at which *g*(*temp*; *predictors*) = logit(50%) = 0. Thus, the crossover point is:

$$\begin{array}{l} 0=intercept\left[predictors\right]+slope\left[predictors\right]\cdot t\left(predictors\right)\\ t\left(predictors\right)=\frac{-intercept\left[predictors\right]}{slope\left[predictors\right]} \end{array}$$

Given the optimal crossover temperature of 33.94°F, we will interpret cases where participants switch from giving to withholding blankets at lower temperatures than 33.94°F as more risky than optimal, as the likelihood of being penalized $6,000 for withholding blankets is higher if the temperature drops below freezing and the alpacas die. We will also interpret cases where participants switch from giving to withholding blankets at higher temperatures than 33.94°F as more conservative than optimal, as the larger penalty ($6,000) is less likely, but it still costs $1,000 to provide blankets. Note that the most conservative strategy would be to issue blankets on each of the 48 trials regardless of the temperature forecast, which would result in $0 remaining in the $48,000 budget.

Using the same procedure described in Experiment 1, we calculated the optimal crossover temperature to change from giving to withholding blankets for each level of variance for Experiment 2. For a distribution with Low-variance, the optimal crossover temperature is 32.97°F, for Medium-variance the optimal crossover is the same as in Experiment 1, which used distributions that had *SD* = 2°F (33.94°F). For High-variance, the optimal crossover temperature is 34.90°F.

## Experiment 1

As previously detailed, reasoning with subjective descriptions of uncertainty is variable (Budescu et al., 2009, 2012), and it is unclear if and how people will update their judgments with additional information about forecaster confidence. A pessimistic hypothesis would propose that indirect uncertainty expressed as forecaster confidence would be too complicated, overwhelming, or variably interpreted to produce meaningful changes in resource allocation judgments. Participants also may not understand the value of the forecaster confidence information and ignore it altogether. An alternative hypothesis would suggest that participants will incorporate information about forecaster confidence but in suboptimal ways, which could include making highly variable judgments or relying on guessing. A final and relatively optimistic approach would be to hypothesize that participants will reasonably update their judgments with the inclusion of indirect uncertainty, possibly making increasingly conservative judgments as the confidence decreases. We summarize these competing predictions below:

- **Lack of effect prediction**: Participants will not be affected by the forecaster confidence manipulation and, therefore, either always issue or always withhold aid. This strategy could be due to participants ignoring the forecaster confidence information, not doing the task, or making extremely cautious decisions.
- **Increased variability prediction**: The variability in participants’ judgments will increase along with indirect uncertainty.
- **Increased caution prediction**: Participants will make increasingly cautious judgments as more indirect uncertainty is expressed.

Experiment 1 aims to determine which strategy most accurately describes participants’ decision patterns when presented with varying levels of forecaster confidence.

### Statistical Analysis

We used the following R packages for the analysis: *tidyverse* v. 1.2.1(Wickham, 2017; data processing), *brms* v. 2.13.0 (Bürkner, 2017, 2018; Bayesian modeling), and *tidybayes* v. 2.0.3 (Kay, 2020; data processing and visualization). We used a Bayesian multilevel binomial logistic regression to examine how participants’ judgments were influenced by indirect qualitative uncertainty, which was communicated as forecaster confidence. We evaluated the amount of variance in participants’ blanket judgments explained by the mean forecast temperatures shown in the stimuli and the levels of forecaster confidence. The dependent variable was participants’ decisions to give blankets (coded as 1) or withhold blankets (coded as 0). Uncertainty communication was included in the model as a fixed effect predictor (coded such that No indirect uncertainty information was the referent). The mean temperature of the distributions in the stimuli was also included as a fixed effect. We centered temperatures around the optimal crossover temperature (33.94°F) in order to interpret the resultant coefficients as riskier (negative values) or more conservative (positive values) than the optimal strategy for this task. R-markdown code and data for this analysis, and all subsequent analyses, can be found in the supplemental material.^1^

Participant ids were included as a random intercept effect, and both Uncertainty Communication and Optimal-centered Temperatures were included as random slopes [R notation: Blanket Decision ~ Uncertainty Communication + Optimal-centered Temperatures + (Uncertainty Communication + Optimal-centered Temperatures | Participant)]. The model specifications included weakly informative priors centered at 0°F with an SD of 2.5°F. These priors were chosen because the lowest mean temperature in the stimuli was 31.5°F, which is 2.5°F from the optimal crossover temperature (33.94°F). For the reader familiar with statistical significance, we used 95% credible intervals to determine if a predictor reliably accounted for a proportion of variance in participants’ judgments to provide or withhold emergency aid. We interpreted predictors with a credible interval that did not include zero as having a reliable effect on participants’ judgments.

### Results

**Figure 6:**
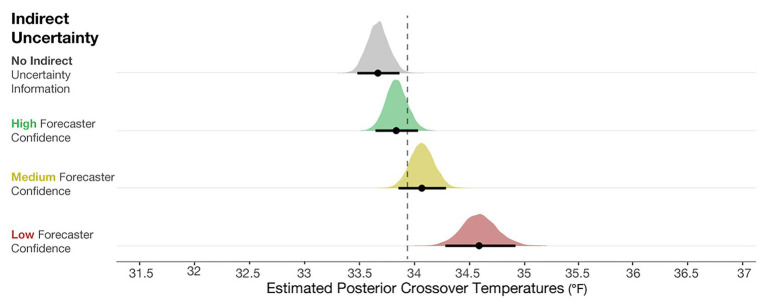
Posterior estimates of the distributions of crossover temperatures for the No indirect uncertainty information condition (block 1), and High, Medium, and Low forecaster confidence conditions (block 2). The black dots represent the posterior mean, and the black lines represent the 95% credible intervals around the means. The dotted line is plotted at 33.94°F, which is the optimal crossover temperature.

To determine if the crossover temperatures meaningfully differed from the optimal crossover temperature (33.94°F), we plotted the posterior distributions for the crossover temperatures of each condition in Figure 6, along with 95% credible intervals (black line) and the means of the distribution (black dot). These observations revealed that judgments in the No indirect uncertainty information condition were riskier than optimal, and those in the Low-confidence condition were more conservative than optimal. These results suggest that when provided with no information about the qualitative uncertainty in a forecast, participants’ judgments were inclined to be riskier than optimal. When provided with forecaster confidence, participants’ judgments were better aligned with the optimal decision and more conservative. In high-risk scenarios in which conservatism is preferable to risk-taking, such as in the real case of emergency aid distribution in Peru, our work suggests that providing risk managers with qualitative indirect uncertainty can have generally positive effects on their decisions.

**Figure 7:**
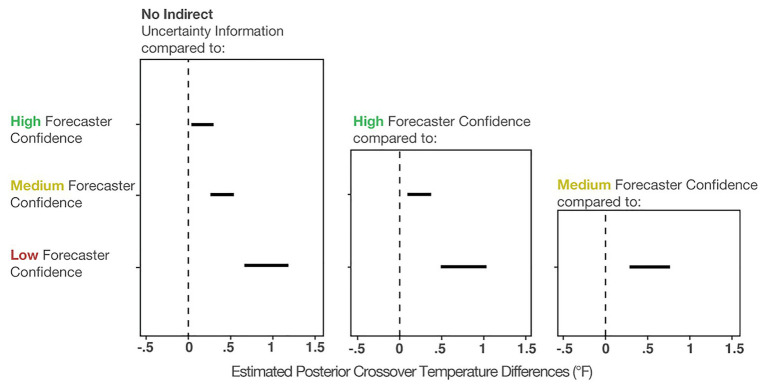
The 95% credible intervals for the comparisons between each forecast confidence condition.

To compare the posteriors of the individual conditions to each other, we plotted the results of the primary analysis in the first column of Figure 7. The findings suggest that when viewing High-, Medium-, and Low-confidence trials, individuals’ crossover temperatures became increasingly more conservative than when no forecaster confidence was presented. The effects became larger when more uncertainty was communicated (e.g., the effect of None-High < None-Medium < None-Low). This result suggests that people make more conservative judgments when they believe uncertainty in a forecast is higher. In sum, these results provide evidence that participants’ judgment patterns are most accurately described by the *increased caution prediction*, which proposed that participants will make increasingly cautious judgments as more indirect uncertainty is expressed.

Figure 7 (middle column) shows the comparisons between High-Medium and High-Low. This analysis revealed that individuals made more conservative judgments when viewing the Low- and Medium-confidence trials compared to High-confidence trials. Finally, Figure 7 (right column) shows that Low-confidence trials elicited more conservative judgments than the Medium-confident trials. In sum, these analyses provide evidence that participants made increasingly conservative judgments when told that the forecasters had less confidence about the forecast.

**Figure 8:**
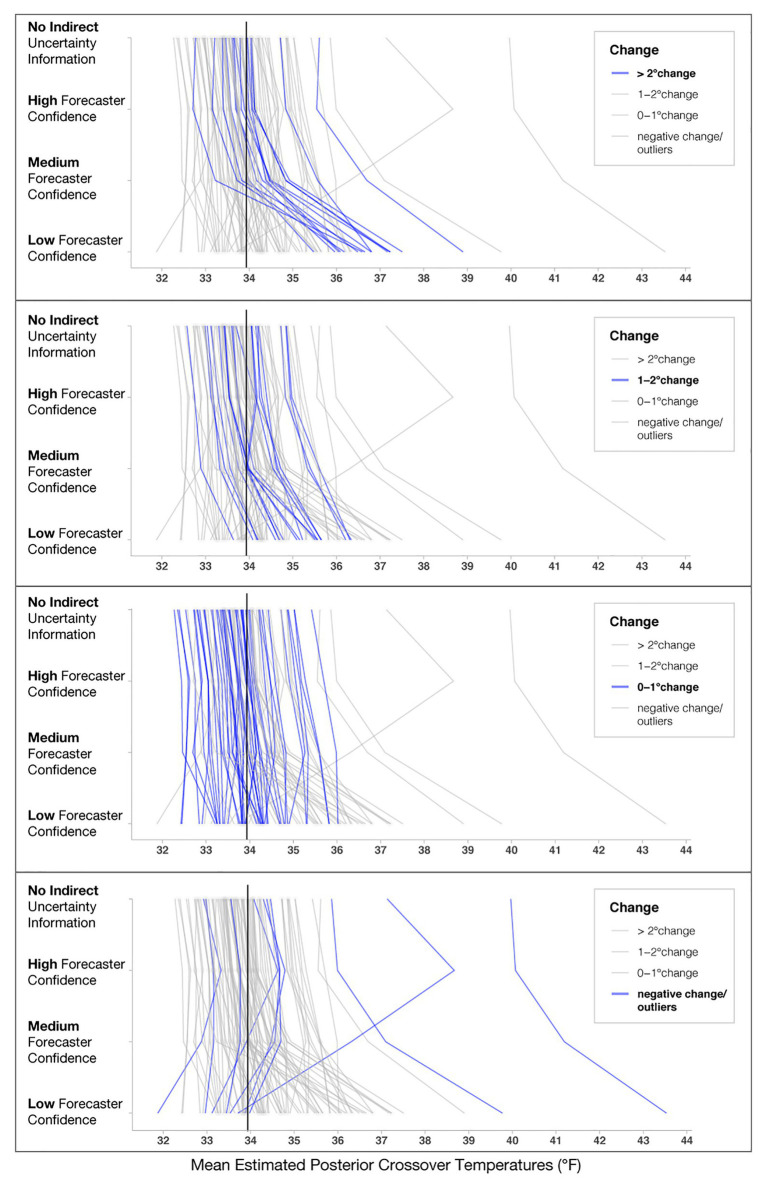
Mean estimated posterior crossover temperatures for each participant in each condition. Each line represents one participant, and the black line represents the optimal crossover temperature. The four panels highlight participants’ decision patterns based on the level of change from No indirect uncertainty information to Low-confidence trials, with the **top panel** highlighting participants who showed the largest impact of forecaster confidence (>2°F) and the **bottom panel** highlighting participants that had negative change or crossovers >39°F, which we considered to be outliers.

In addition to the model’s posterior estimates for each condition, we visualized the crossover temperatures for each person to visually assess possible individual variation present in decision patterns not captured in the analysis of the full data set (see Figure 8). To examine if some participants utilized different strategies, such as ignoring forecaster confidence ratings, we visualized four levels of changes between no forecaster confidence information and low forecaster confidence in the panels in Figure 8. Roughly half of the participants (*n* = 46) showed only small changes across the conditions (0–1°F), with 19 showing changes between 1 and 2°F and 17 showing the largest changes (>2°F). Interestingly, those demonstrating the largest changes seemed to respond dramatically to trials with low forecaster confidence. The crossover temperatures of these individuals increased on average, 2.25°F from Medium- to Low-confidence trials, whereas those with more moderate changes (e.g., 1–2°F) increased their crossovers by only 0.91°F on average. Observationally, the decay is more rapid for those in the >2°F group. For those in the 1–2°F group, the decay appears to be linear.

**Figure 9:**
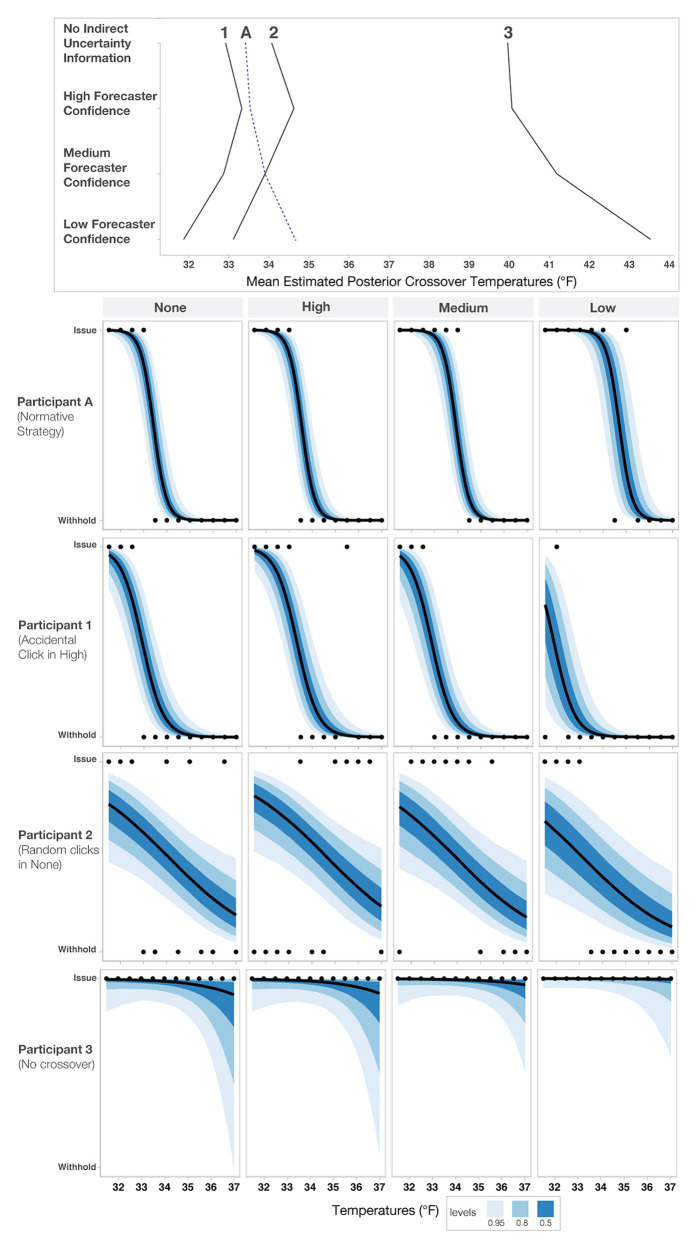
Sensitivity analysis of three outlier responses (Participants 1–3) and an increased caution decision pattern (Participant A). The **top panel** shows each participant’s crossover pattern for each condition. The **lower panels** show each participant’s raw response pattern, which indicates the choice to either issue or withhold aid. The black dots represent each participant’s judgment for a given temperature, and the blue bands represent credible intervals.

Several individuals in the “negative change/outliers” group had a negative change in either their crossovers or crossover temperatures >39°F (*n* = 8; bottom panel of Figure 8). To investigate why the model produced extreme values for these participants, we visualized responses for a subset of them (see Figure 9). Figure 9 shows that for this subset of participants, the extreme values are due to a combination of inconsistent responses in some conditions but not others. For example, Participant 1 decided to give blankets in the High-confidence condition at 35.5°F, which is inconsistent with their pattern for other levels and might have been an accident. Participant 2 decided to issue and withhold blankets when provided no information about uncertainty, a strategy that appears inconsistent with their decisions in the other conditions. Participant 3 always issued blankets regardless of the information in the stimuli, which may be due to ignoring the confidence information or an overly cautious strategy. As a point of reference, we also included Participant A, who used the more common *increased caution* decision pattern.

To determine if these individuals negatively impacted the overall model’s fit, we excluded them from the model and reran the primary analysis. The effects of the second model were slightly larger than the original model but not meaningfully different (e.g., the conclusions and magnitude of the effects did not change). Therefore, we decided to report the original findings to be conservative, and thus all the participants and all responses are included in the analysis reported throughout the paper.

**Figure 10:**
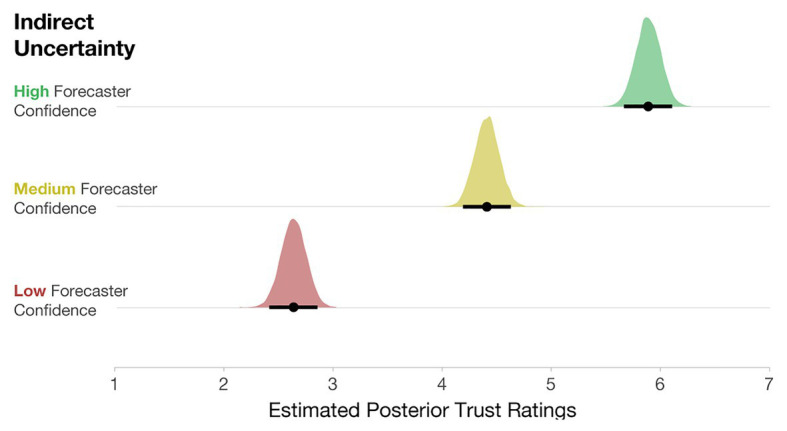
Posterior estimates of the distributions of trust ratings for each condition. Black dots represent posterior means, and black lines represent 95% credible intervals.

After completing the initial experiment, participants also rated the trustworthiness of the High-, Medium-, and Low-confidence stimuli. To evaluate the impact of forecaster confidence on trust ratings, we computed a Bayesian multilevel linear regression. Trust ratings were predicted by forecaster confidence as a fixed effect, and Participant was included as a random intercept [e.g., Trust ~ Uncertainty Communication + (1| Participant)]. The model specifications included weakly informative priors centered at 3.5 (halfway between the Likert scale from 1 to 7) with an SD of 2. As seen in Figure 10, participants rated Low-confidence forecasts as the least trustworthy, Medium- as more trustworthy, and High-confidence forecasts as the most trustworthy.

### Discussion

Regarding the predictions proposed in the experimental overview, the results of this study suggest that participants utilize indirect qualitative uncertainty when conveyed as quantile dotplots and forecaster confidence. In manipulations in which forecasters were less sure about their prediction, participants made more conservative judgments. These results are likely due, in part, to our use of quantile dotplots, which are proving to be consistently more effective than many other communication techniques of uncertainty (Kay et al., 2016; Fernandes et al., 2018). The majority of studies on uncertainty visualization have used variants of error bars (e.g., Belia et al., 2005; Correll and Gleicher, 2014; Scown et al., 2014), which might account for some of the seemingly poor decision-making. Further, we found that when provided with no information about forecaster confidence, participants’ decisions were riskier than optimal. In light of these findings, we encourage visualization designers to consider the implications of their viewers’ decisions. If erring on the side of caution is desirable, as is the case with many hazard forecasts, then the results of this study suggest that communicating forecaster confidence is appropriate.

The statistical analysis provided support for the caution strategy, but individual response patterns also indicated that some people always issue or withhold blankets. It is difficult to speculate about what accounts for the lack of an impact of forecaster confidence. Some possibilities include participants ignoring the forecaster confidence information, not doing the task, or making extremely cautious decisions. Additionally, the prediction that people make more variable judgments with higher uncertainty has some support. As seen in both the posterior distributions (Figure 6) and the individual response patterns (Figure 8), judgments in the Low-confidence trials are more variable. The findings suggest that an increased cautiousness strategy may drive participants’ judgments, but when told that a forecaster has low confidence in a prediction, participants’ responses become more variable.

Lastly, participants’ trust ratings corresponded reasonably well with forecaster confidence, where Low-confidence trials received the lowest trust ratings, with High-confidence trials receiving the highest trust ratings. These results are not that noteworthy; however, it will be interesting to see how trust ratings are impacted when variance information is also included in Experiment 2. Experiment 1 demonstrates the influence of forecaster confidence only in relation to forecasts that never changed in variability. In Experiment 2, we seek to expand our understanding of how people reason with direct and indirect uncertainty by examining cases where the variability of the forecast changes along with the levels of forecaster confidence.

## Experiment 2

In Experiment 2, we tested whether participants can update their judgments to account for changes in variability shown in quantile dotplots (e.g., Low-variance: *SD* = 1°F, Medium-variance: *SD* = 2°F, High-variance: *SD* = 3°F). People may use a variety of strategies when attempting to reason with direct and indirect uncertainty. Experiment 2 aims to identify which of the following decision patterns most accurately describes participants’ resource allocation judgments with direct and indirect uncertainty.

- **Lack of effect prediction**: Participants will not be affected by the variance manipulation, the forecast confidence manipulation, or both.
- **Increased variability prediction**: The variability in participants’ judgments will increase proportionally to the variance displayed in the quantile dotplots and indirect uncertainty.
- **Increased caution prediction**: Participants will make increasingly cautious judgments along with increases to the variance of the quantile dotplots and indirect uncertainty. For example, participants may increase the conservatism of their judgments as the quantile dotplots display distributions with a higher variance. Further, conditional on variance, participants’ judgments will be more conservative as indirect uncertainty increases.
- **Saliency prediction**: Participants’ judgments will be impacted primarily by the variance information as the variance is communicated within the quantile dotplot and, therefore, likely will be more visually salient than the forecaster confidence communicated in text.
- **Conflict prediction**: Participants’ judgments will vary predictably with the variance and forecaster confidence manipulations, except for situations where there is a conflict between the direct and indirect uncertainties. For example, participants may consider distributions with low-variance to be relatively reliable. However, if participants are also told the forecasters have low confidence in the forecast with low variance, the information represented in the quantile dotplot (variance) and the text (forecaster confidence) are in opposition. In cases of conflict between direct and indirect uncertainty, the normative response pattern may change, and judgments may become more variable or overly conservative.

To examine how participants integrate direct quantitative uncertainty and indirect qualitative uncertainty, we replicated the design of Experiment 1, but we changed the quantile dotplots to include Low-, Medium-, and High-variance.

### Statistical Analysis

We used the same Bayesian multilevel logistic regression approach as in Experiment 1 to determine the influence of forecaster confidence and variance on participants’ decisions to give or withhold blankets. The dependent variable in the model was the participants’ decision to give blankets (coded as 1) or withhold blankets (coded as 0). The fixed effect predictors included Uncertainty communication (coded such that No indirect uncertainty information was the referent), Variance (coded such that Low-variance was the referent), the interaction between Uncertainty Communication*Variance, and the optimal-centered mean temperature of the distributions in the stimuli. We centered temperatures around the optimal crossover temperature for each level of variance to account for the differences in optimal crossovers associated with each variance. Said another way, for dotplots with Low-variance, we subtracted 32.97 from each of the temperatures; for dotplots with Medium-variance, we subtracted 33.94, and for High-variance we subtracted 34.90. The resultant optimal-centered crossovers can be interpreted as zero being the optimal crossover, accounting for each variance, with negative values as more risky and positive values as more conservative than optimal. Participant ids were included as random intercept effects. We included the following as random slopes in the model: Optimal-centered Temperatures, the interaction between Uncertainty Communication*Variance, and the lower order terms of the interaction [e.g., R notation: Blanket Decision ~ Uncertainty Communication*Variance + Optimal-centered Temperatures + (Uncertainty Communication*Variance + Optimal-centered Temperatures | Participant)]. In line with the approach used in Experiment 1, the model specifications included weakly informative priors centered at 0 with an *SD* = 2.5.

### Results

**Figure 11:**
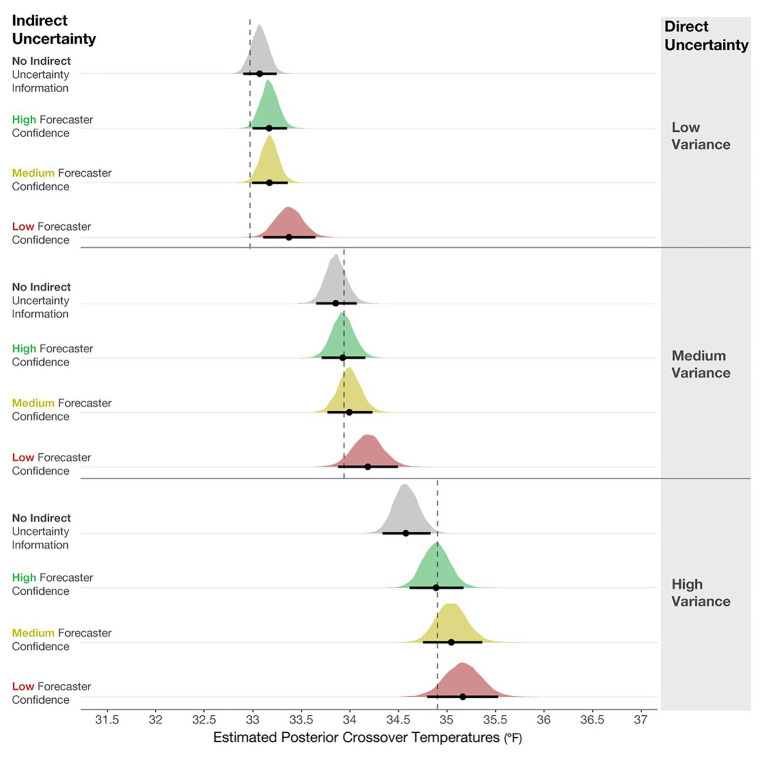
Posterior estimates of the distributions of crossover temperatures for Low-, Medium-, and High-variance conditions, and the High-, Medium-, Low-confidence, and No indirect uncertainty information conditions. The black dots represent the posterior means, and the black lines represent the 95% credible intervals around the means. The dotted lines are optimal crossover temperatures for each level of variance.

The posterior distributions of the crossover temperatures are shown in Figure 11, which demonstrates that participants’ judgments became increasingly conservative when the variance increased, which provides support for the *increased caution prediction* (i.e., mean crossover temperature for Low- < Medium- < High-variance). The increased conservatism in participants’ resource allocation judgments is in line with the optimal crossover temperatures for each variance level. This finding suggests that participants can relatively accurately incorporate direct uncertainty communicated in quantile dotplots into their judgments. Consistent with Experiment 1, the results indicate that participants also made increasingly conservative judgments when the indirect qualitative uncertainty increased. The impact of forecaster confidence was not as large as in Experiment 1, but it was in the same direction. Given that the participants were presented with quantile dotplots with highly salient differences in their SDs, they possibly focused more on the visual depiction of uncertainty (variance) than the textual expression of uncertainty (forecaster confidence). Our data suggest that the impacts on participants’ decisions were driven more by the variance manipulation than the forecaster confidence manipulation, adding support to the *saliency prediction*.

**Figure 12:**
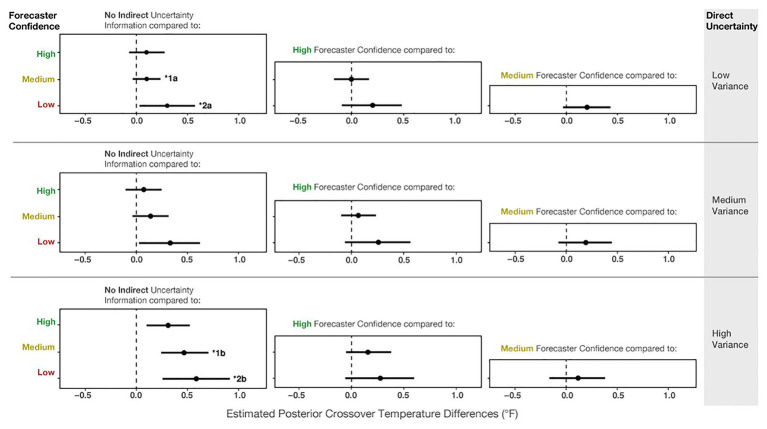
The 95% credible intervals for the comparisons between each forecast confidence condition.

Even though the effect of variance was dominant in Experiment 2, our results suggest that people incorporated the forecaster confidence information into their judgments, which is revealed in both main effects and two reliable interactions in the model results. Figure 12 shows the mean posterior distributions and credible intervals for all the comparisons in the model. For Low-, Medium- and High-variance, participants made reliably more conservative judgments with Low forecaster confidence compared to No communication of confidence (see Figure 12 left column). For High-variance, participants also made more conservative judgments with Medium and High forecaster confidence compared to No communication of confidence. Experiment 2 suggests that participants’ judgments were influenced by forecaster confidence ratings but not as pervasively as in Experiment 1.

We also found two reliable interactions in the model. The first is between No communication vs. Medium confidence and Low-variance vs. High-variance (*b* = 0.94, *CI* = [0.39, 1.49]; superscript 1) in Figure 12. This interaction suggests that for High-variance, participants made more conservative judgments with Medium forecaster confidence compared to when no uncertainty was communicated (Figure 12, superscript 1b). In contrast, for Low-variance, we did not observe such a difference between No communication and Medium forecaster confidence (superscript 1a). The second interaction (*b* = 0.72, *CI* = [0.09, 1.37]) revealed that for Low-variance, conservatism showed a small increase from No communication of uncertainty to Low forecaster confidence (Figure 12, superscript 2a), and this relative increase in conservatism was larger for High-variance (Figure 12, superscript 2b). In sum, the interactions suggest that for High-variance, when participants received no information about forecaster confidence, they made riskier decisions than optimal – further, the increased conservatism from the No communication of uncertainty to Medium and Low forecaster confidence trials was greater for High-variance than for Low-variance.

**Figure 13:**
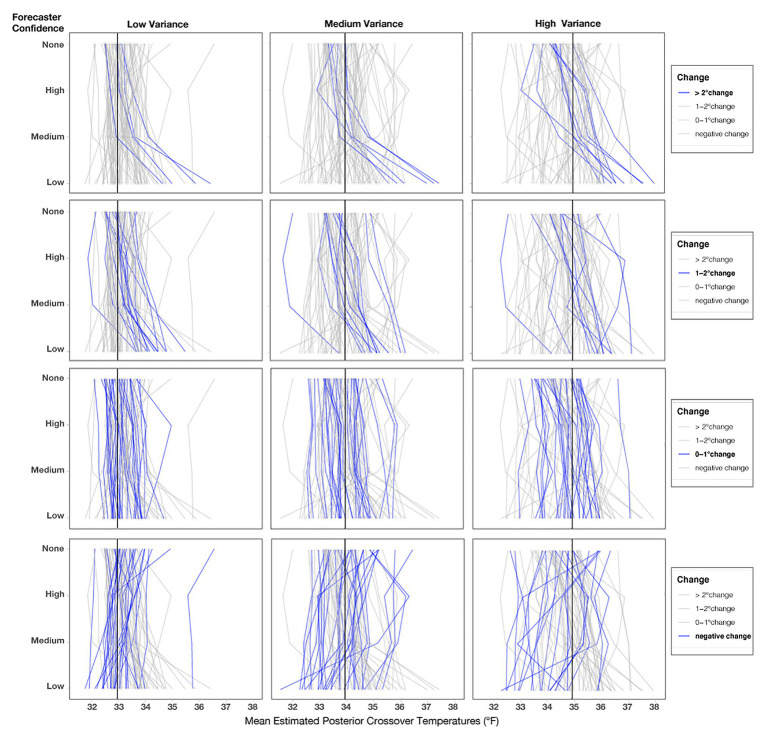
Mean posterior crossover temperatures for each participant in each condition. Each line represents one participant, and the black line represents the optimal crossover temperature for each variance condition. The three columns represent the variance conditions, and the four rows highlight participants’ decision patterns based on the level of change from No indirect uncertainty information (None) for Low-confidence trials.

As in Experiment 1, we also visualized the crossover temperatures for each participant to evaluate how an individual’s judgments changed across the conditions. Before visualizing Figure 13, we investigated outliers, including participants who did not have crossovers and those with crossover values >39°F or <32°F. We found that the outlier crossovers were due to the same issues detailed in Experiment 1, illustrated in Figure 9. These participants had only one response (e.g., always giving or withholding aid), made random patterns of decisions, or seemed to make an accidental click. The numbers of participants in Experiment 2 who had no crossovers in each condition for Low-variance (None = 6.6%, High = 4.4%, Medium = 2.2%, Low = 7.7%) and Medium-variance (None = 3.3%, High = 4.4%, Medium = 2.2%, Low = 7.7%) were relatively similar. However, for the High-variance trials, participants made more outlier responses when told that the forecasters had Low confidence (None = 5.5%, High = 8.8%, Medium = 8.8%, Low = 14.4%). The higher number of outliers for the High-variance condition with Low forecaster confidence provides evidence for the *lack of effect prediction*. Again, assigning causality to such data is pure speculation, but it is interesting that outliers increased for the condition with the highest combined uncertainty.

We reran the primary model for Experiment 2 without two participants who never changed their judgments across any of the conditions, and the findings did not meaningfully differ from the results of the previous report. As seen in Figure 13, roughly one-third of participants showed small changes (0–1°F) from No Communication of forecaster confidence to Low forecaster confidence across each variance condition (Low = 31%, Medium = 32%, High = 32%). Fewer participants showed changes between 1 and 2°F (Low = 16.6%, Medium = 13.3%, High = 16.6%), and the fewest showed changes >2°F (Low = 11.1%, Medium = 13.3%, High = 18.8%). Surprisingly, a large number of people showed a risky response pattern (e.g., a negative value when comparing their crossover temperature for No communication to Low-confidence; Low = 41%, Medium = 41%, High = 32%). These negative response patterns were not entirely due to outlier responses, which we validated by visualizing individuals’ crossovers after removing outliers for each level of variance, as seen in Figure 13. We defined outliers as crossover values <32 or >39°F, which were removed only for Figure 13 (number of outliers removed; Low = 16, Medium = 14, High = 23). Response patterns in Experiment 2 were more variable than in Experiment 1. Further, note that the spread of response patterns in Figure 13 is larger for High > Medium > Low across all conditions. The additional variability in response patterns, which corresponds to the increases in variance, provides support for the *increased variability prediction*.

**Figure 14:**
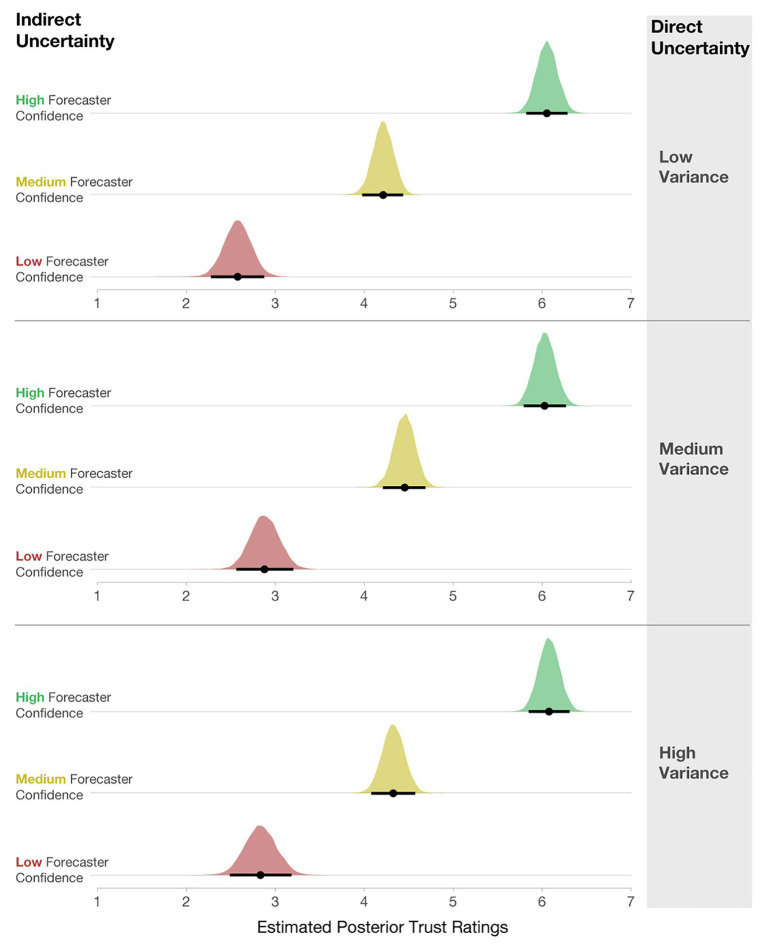
Posterior estimates of the distributions of trust ratings for each condition. The black dots represent posterior means, and black lines represent 95% credible intervals.

Lastly, we also analyzed how participants’ ratings of trust were influenced by variance and forecaster confidence. We computed a Bayesian multilevel linear regression, where trust ratings were predicted by forecaster confidence and variance as a fixed effect, along with their interaction. Participant ids were included as a random intercept, and the interaction of Uncertainty Communication and Variance was included as a random slope [e.g., Trust ~ Uncertainty Communication*Variance + (Uncertainty Communication*Variance | Participant)]. The model specifications included weakly informative priors centered at 3.5 with an *SD* = 2. As seen in Figure 14, participants rated Low-confidence forecasts as the least trustworthy, Medium- as more trustworthy, and High-confidence forecasts as the most trustworthy. These findings exhibit the same pattern as in Experiment 1.

Interestingly, the level of variance had little effect on participants’ interpretation of trust. We found no meaningful main effects of variance in the model output. Two minimal interactions (Low- vs. High-confidence x Low- vs. Medium-variance; *CI* [0.05, 0.61] and Low vs. Medium-confidence x Low- vs. Medium-variance; *CI* [0.02, 0.52]) do not warrant further investigation. This finding suggests that when considering trust, qualitative statements about forecaster confidence may impact trust more than quantified variability in a model that is shown visually. Recent prior work finds that linguistic expressions of direct uncertainty (e.g., “484,000 people in the UK were unemployed …there is some uncertainty around this estimate, it could be somewhat higher or lower”) evoke less trust than numeric expressions (e.g., “…484,000 people in the UK were unemployed…minimum 1,413,000 to maximum 1,555,0000”; van der Bles et al., 2020). Similarly, the current findings show that linguistic expressions of forecaster confidence have a larger impact on trust ratings than direct quantitative uncertainty.

### Discussion

The critical finding from Experiment 2 was that participants made more conservative judgments when both the direct quantitative uncertainty (variance) and indirect qualitative uncertainty (forecaster confidence) were increased. Consistent with Experiment 1, judgments made from no expression of forecaster confidence were the riskiest, and communicating more indirect and direct uncertainty led to more conservative judgments. Of particular interest is the finding that when participants did not receive forecaster confidence information and viewed quantile dotplots with High-variance, their judgments were riskier than optimal. These findings suggest that applications where forecasts have high variability, such as long-term future forecasts of climate predictions, could benefit from the addition of qualitative uncertainty estimates.

Between the two types of uncertainties, direct quantitative uncertainty (variance) had a more substantial impact on participants’ resource allocation decisions. A variety of reasons explain why the direct quantitative uncertainty may have had a larger impact on participants’ judgments, including that direct uncertainty was shown in the visualization, and the indirect uncertainty was shown in the text. Participants might have spent longer considering the direct uncertainty because the quantile dotplot may have been more visually salient, which could explain the prominence of the direct uncertainty in their judgments. Future work could identify which information participants focused on using eye-tracking measures.

Additionally, we found more variability between and within participants’ judgments in Experiment 2 compared to Experiment 1. We do not know if the increased variability is due to the complication of reasoning with two types of uncertainty or if the experiment was overwhelming for the participants as the information varied on multiple dimensions. Interestingly, some of the most variable responses were in the highest combined uncertainty condition (High-variance + Low forecaster confidence). Additional research is needed to carefully examine the source of the increased variability in Experiment 2.

The impact of forecaster confidence was not as large as in Experiment 1, and we saw less discrimination between the confidence conditions. The pattern, however, was in the same direction as in Experiment 1. For all variance levels, the Low-confidence conditions elicited more conservative judgments than when No forecaster confidence was presented. Distributions with High-variance and Low, Medium, and High forecaster confidence elicited more conservative judgments than trials with High-variance and No information about forecaster confidence. The reduction in the effect of forecaster confidence may be due to the salience of the variance information, or that participants were more confused by the various types of information presented. Given that individuals’ response patterns were considerably more variable in Experiment 2 compared to Experiment 1, participants may have been more overwhelmed with the multiple types of uncertainties. More work is needed to determine the source of the variability in participants’ response patterns in Experiment 2.

## General Discussion

The two experiments presented in this paper provide new evidence that people can and do utilize both direct quantitative and indirect qualitative uncertainty in resource allocation decisions. In the first experiment, we found that participants made increasingly conservative judgments when they learned the forecasters had less confidence in nighttime low-temperature forecasts. In the second experiment, we found that when presented with both forecaster confidence and forecast variance represented in quantile dotplots, participants incorporated both types of information into their decisions. When participants were shown distributions of forecasted nighttime low temperatures that had higher variance, they made more conservative judgments, and when told the forecasters were less sure about the forecasts, participants also made more conservative judgments. We found that the variance in the visualized distributions had a more substantial effect on participants’ resource allocation judgments than the forecaster confidence information. Together, these studies provide insights into how people conceptualize both direct and indirect uncertainty within a single forecast using best practices in visualization research.

The presented results propose that it is possible for people to reason with multiple types of complex uncertainty information, which is a human reasoning capacity that has had inconsistent empirical support. Science communicators report being worried that uncertainty may be too complicated for the average person to understand (Fischhoff, 2012; Gustafson and Rice, 2019; Hullman, 2019). Various studies demonstrate that when presented with uncertainty in some textual formats (e.g., Kahneman and Tversky, 1979, 1982; Baron, 1997) and in some visualizations (e.g., Belia et al., 2005; Joslyn and LeClerc, 2013; Correll and Gleicher, 2014; Padilla et al., 2015, 2020; Ruginski et al., 2016; Hofman et al., 2020), people make errors that reveal their misunderstandings of the data. Most famously, Belia et al. (2005) found that highly educated scientists and medical professionals misunderstand how to interpret error bars, which are one of the primary ways researchers communicate statistical results in their disciplines. Further, Joslyn and LeClerc (2013) found that participants misunderstand error bars for a nighttime low-temperature forecast, and the misunderstanding remained even when the authors included a key that described how to interpret the visualization. Other work finds that misunderstandings of uncertainty visualizations persist after extensive training on how the scientists created the visualizations and how to interpret them correctly (Boone et al., 2018; Padilla et al., 2020).

The substantial body of research demonstrating how difficult it can be to reason with one form of uncertainty makes the current finding that people can effectively make decisions with two types of uncertainty more noteworthy. In an extension of perspectives proposed by Gigerenzer and Hoffrage (1995), we suggest that visualizations can support reasoning with highly complex data when the visualizations are designed using intuitive formats and best visualization practices. In particular, quantile dotplots have produced consistently positive results when used to express uncertainty in distributional data (Kay et al., 2016; Hullman et al., 2017; Fernandes et al., 2018; Greis et al., 2018).

This work provides promising evidence that people can relatively accurately incorporate multiple types of uncertainty into their judgments, but this research has some caveats and remaining open questions. In the present studies, a confound exists between the communication format and different types of uncertainties, making it impossible to attribute the current findings to the communication format or uncertainty type alone. For example, additional research is needed to determine if participants were more influenced by direct quantitative uncertainty because it was visually displayed in quantile dotplots or because quantitative uncertainty is more influential in their decisions. A similar confound exists for the interpretation of the trust ratings. For example, it is unclear if levels of direct uncertainty do not influence participants’ beliefs about trust or if this finding is due to the direct uncertainty being expressed with a visualization.

Future research may wish to expand on the variety of limitations to the current studies. Scholars have documented issues with the categorical expert expressions of confidence used by the IPCC (Budescu et al., 2009, 2012) and others (for review, see O’Hagan et al., 2006). Budescu et al. (2012) found that using a dual categorical-numeric scale can decrease the variability in participants’ interpretations of categorical forecaster confidence. We elected to adhere to the definition of indirect uncertainty defined in the background section (i.e., indirect uncertainty cannot be quantified explicitly; Der Kiureghian and Ditlevsen, 2009), which is why we used the categorical expression of forecaster confidence rather than the approach proposed by Budescu et al. (2012). We considered this to be a first step in determining if people could reason with multiple forms of uncertainty from a single source, and, therefore, we opted not to complicate the stimuli with both categorical and numeric expressions of forecaster confidence. Further, future work may want to test alternative methods for communicating forecaster confidence in both text and visualizations. Some of the additional variability observed in Experiment 2 may be due to the experiment’s within-subjects design. We selected a within-subjects design explicitly to determine how participants’ judgments changed in response to our manipulations, but we acknowledge this design choice has pros and cons. We presented participants with many stimuli that had various manipulations, which may have contributed to the increase in variability in Experiment 2. Researchers may want to consider a between-subjects design to determine if increased variability in judgments is genuinely a property of how people reason with direct and indirect uncertainty.

Beyond the limitations within the design of the study, we need to consider the ecological, external, and construct validity of visualization research (Padilla, 2018). Ecological validity is how closely the experiment matches real-world tasks (Chaytor and Schmitter-Edgecombe, 2003). In the current study, we selected the task’s context based on a real decision scenario in the humanitarian sector. However, the costs and payoffs were based on prior uncertainty visualization research, which does not reflect the real-world context. When gains and losses are changed, people may demonstrate more or less of an effect than documented in the current work. Considering external validity (e.g., the capacity to generalize the finding to other contexts and groups; Berkowitz and Donnerstein, 1982), we argue that most people will demonstrate the same decision pattern found here. Participants on Amazon’s Mechanical Turk are diverse and have a range of skill sets, which offers an appropriate baseline. However, individuals with more expertise in visualization, statistics, or crisis management may employ different strategies. Further, we suspect that these findings will be consistent with future visualization studies but might not directly simulate real-world contexts, which may have additional constraints, such as time pressure and distraction. Finally, in terms of construct validity (e.g., the ability of the work to measure what it claims to measure; Cronbach and Meehl, 1955), we took measures to ensure that our findings are an accurate reflection of how people reason with direct quantitative uncertainty and indirect qualitative uncertainty. For example, participants completed the same task numerous times to ensure test-retest reliability. In summary, more empirical work is needed to determine if the current findings can be generalized to real-world contexts, with viewers of various expertise and task constraints, but we argue this work has high construct validity.

The current work analyzed the results of our study statistically, and our future work aims to uncover potential latent processes that guide individuals’ decision strategies when reasoning with direct and indirect uncertainty. We seek to extend the current study by developing a mathematical framework (e.g., Mendoza and Gutiérrez-Peña, 2010; Aliev, 2013) to model the direct and indirect uncertainty decision-making process. Individuals likely develop and implement implicit strategies when making decisions with direct and indirect uncertainty, and variations in these strategies affect each decision’s utility (i.e., the decision’s psychological and monetary reward structure). Our future work seeks to incorporate decision theory to identify the optimal process for incorporating direct and indirect uncertainty to maximize a participant’s utility. Modeling our study in this way is fitting and can provide additional insight into such processes that statistical analyses alone may not permit. Decision theory would thus allow for the robust and detailed modeling of mental integration of direct and indirect uncertainty and would give us insight into how individuals use their conceptualization of both uncertainties to make decisions.

## Conclusion

Reasoning with uncertainty is challenging, and making decisions with multiple forms of uncertainty increases the complexity of an already difficult task. Nonetheless, to ensure accuracy and transparency, scientists in disciplines that produce forecast models require a method for communicating uncertainty that can be quantified directly and uncertainty associated with the accuracy of their models. Before this work, we had no clear indication of how people mentally combine visual and textual representations of direct and indirect uncertainty within a forecast. Our work’s findings provide empirical evidence that participants update their judgments in the direction predicted by both qualitative confidence information (e.g., becoming more conservative when the forecaster confidence is low) and quantitative uncertainty (e.g., becoming more conservative when the variance is increased). Our results lead us to recommend that forecasters present qualitative expressions of model confidence whenever possible alongside quantified uncertainty. We propose that the apprehension of communicating uncertainty in science can be assuaged if science communicators use best practices in their uncertainty communication.

## Data Availability Statement

The datasets presented in this study can be found in online repositories. The names of the repository/repositories and accession number(s) can be found at: https://osf.io/atr57.

## Ethics Statement

The studies involving human participants were reviewed and approved by Northwestern University IRB. The patients/participants provided their written informed consent to participate in this study.

## Author Contributions

LP led the writing of this article and project development. MP contributed to the writing and development of the modeling. MK contributed to the development of the experiment, statistical analysis, and interpretations of the findings. JH provided senior-level guidance and mentorship on all aspects of this work. All authors contributed to the article and approved the submitted version.

### Conflict of Interest

The authors declare that the research was conducted in the absence of any commercial or financial relationships that could be construed as a potential conflict of interest.

## Footnotes

^1^Supplemental materials are stored on the Open Science Framework: https://osf.io/atr57/?view_only=6d2c31aa499d490db70498530a06cad4
